# Supplementary material for: Strongly anharmonic flux-tunable transmon based on InAs-Al 2D heterostructure
Source: Nat Commun. 2025 Dec 15;17:740. doi: 10.1038/s41467-025-67420-1 (PMC12820170; doi:10.1038/s41467-025-67420-1)
Supplement: Supplementary file 1 — Supplementary Information [file 41467_2025_67420_MOESM1_ESM.pdf]

Supplementary Information for strongly anharmonic flux-tunable transmon based on InAs-Al 2D heterostructure

Shukai Liu[1,*], Arunav Bordoloi[1,*], Jacob Issokson[2], Ido Levy[2], Maxim Vavilov[3], Javad Shabani[2], and Vladimir Manucharyan[1,4]

[1]Department of Physics, Joint Quantum Institute, and Quantum Materials Center, University of Maryland, College Park, MD, USA
[2]Center for Quantum Information Physics, Department of Physics, New York University, New York, NY, USA
[3]Department of Physics, University of Wisconsin-Madison, Madison, WI, USA
[4]Institute of Physics, Ecole Polytechnique Federale de Lausanne, Lausanne, Switzerland
[*]These authors contributed equally to this work.

Supplementary Information 1: Experimental setup

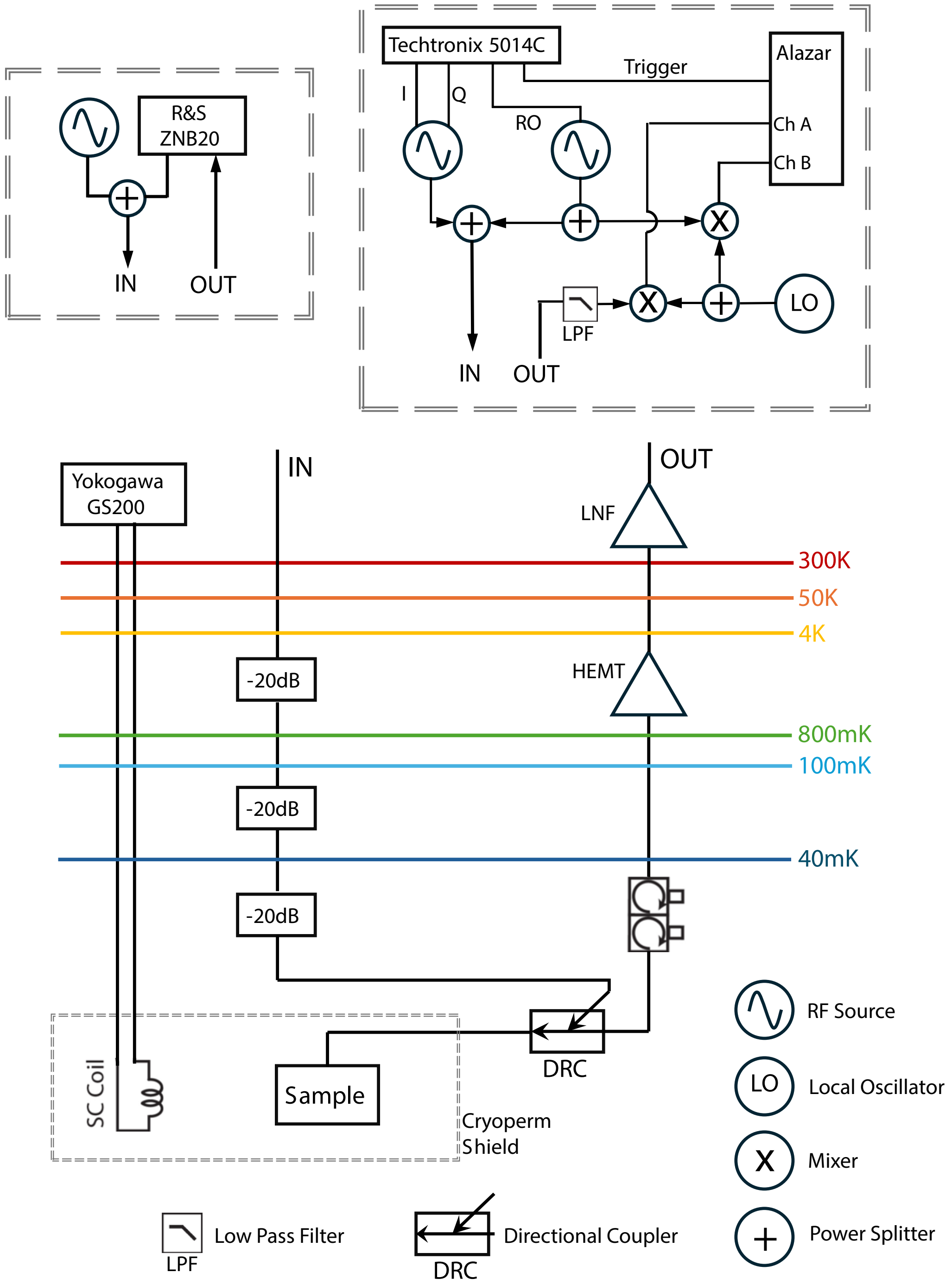

Supplementary Figure 1: Schematics of the experimental setup use for measuring the flux-tunable gatemon qubit.

Supplementary Information 2: One-Tone Spectroscopy

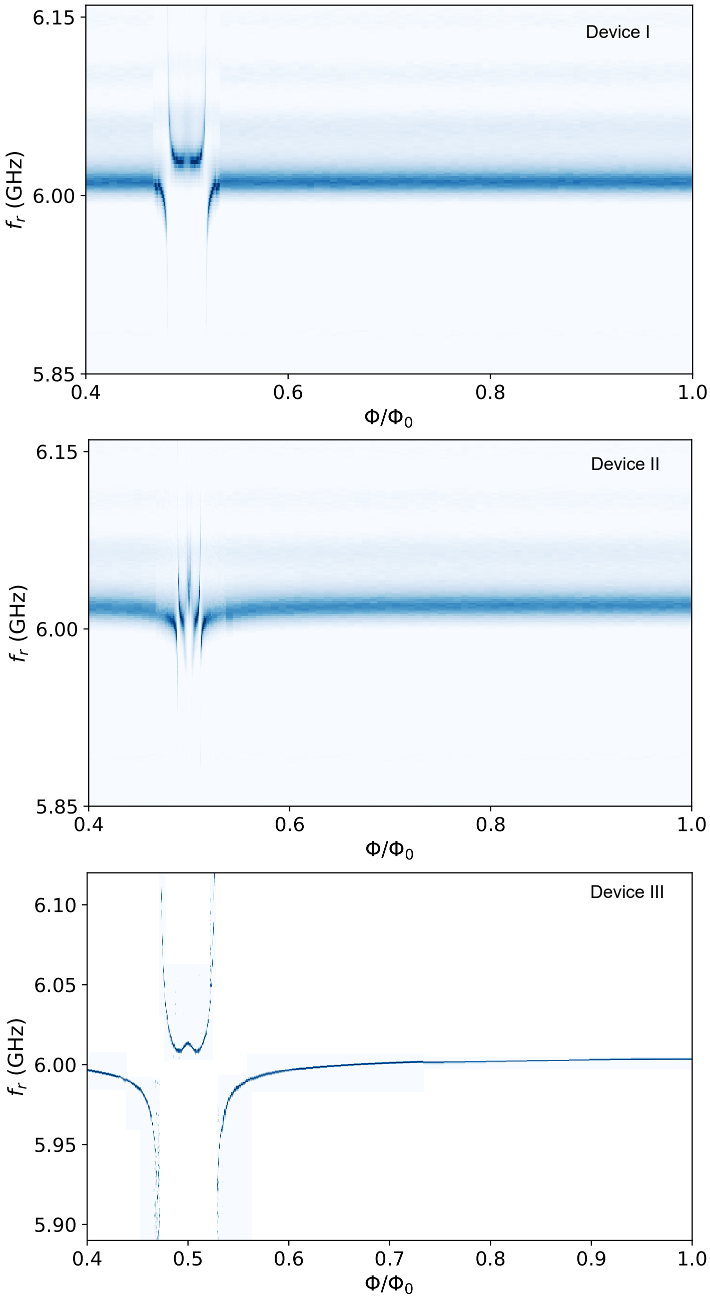

Supplementary Figure 2: One-tone spectroscopy showing $|S_{11}|$ as a function of the resonator drive frequency f_r and the applied external magnetic flux Φ/Φ_0 for device I, II and III, respectively. The resonator response exhibits a vacuum Rabi splitting with a cavity-qubit coupling strength of $g = 122\,\mathrm{MHz}$ for device I, $g = 101\,\mathrm{MHz}$ for device II and $g = 170\,\mathrm{MHz}$ for device III, respectively.

Supplementary Information 3: First-order flux insensitivity at $\Phi = 0.5\,\Phi_0$

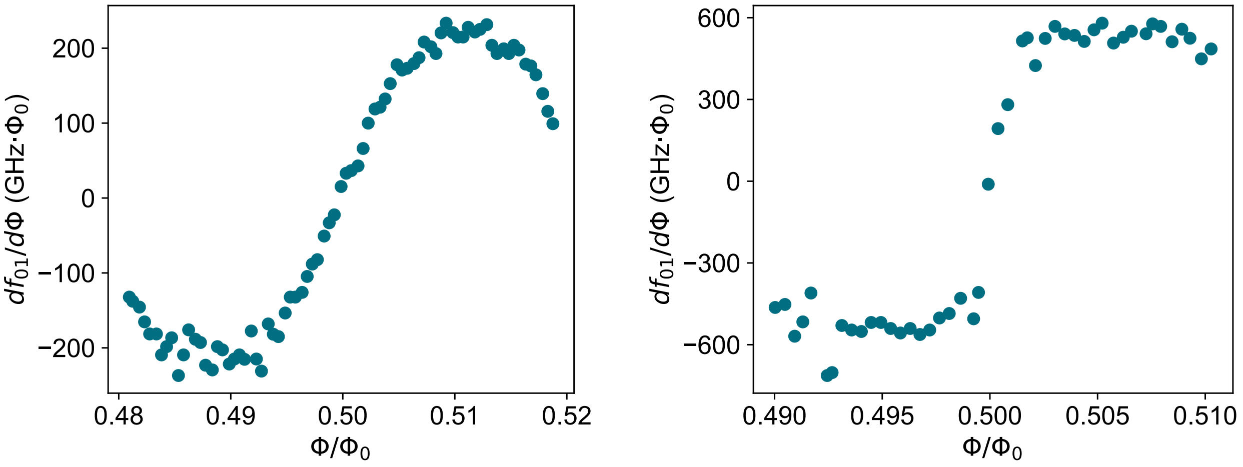

Supplementary Figure 3: Derivative of the $|0\rangle - |1\rangle$ transition qubit frequency $\mathrm{d}f_{01}/d\Phi$ as a function of the applied external magnetic flux Φ/Φ_0 for device I (left) and device II (right), indicating a first-order insensitivity to flux at the half-flux quanta $\Phi = 0.5\,\Phi_0$.

Supplementary Information 4: Charge-matrix elements

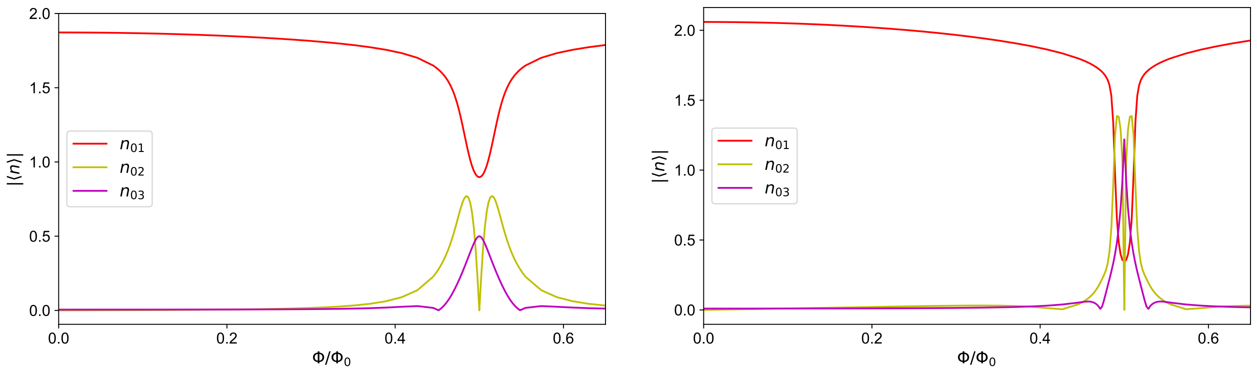

Supplementary Figure 4: Charge matrix elements vs flux Φ/Φ_0 for device I (left) and device II (right) calculated using the single-characteristic-transparency model fit of the device spectra.

Supplementary Information 5: Higher-harmonic model comparison

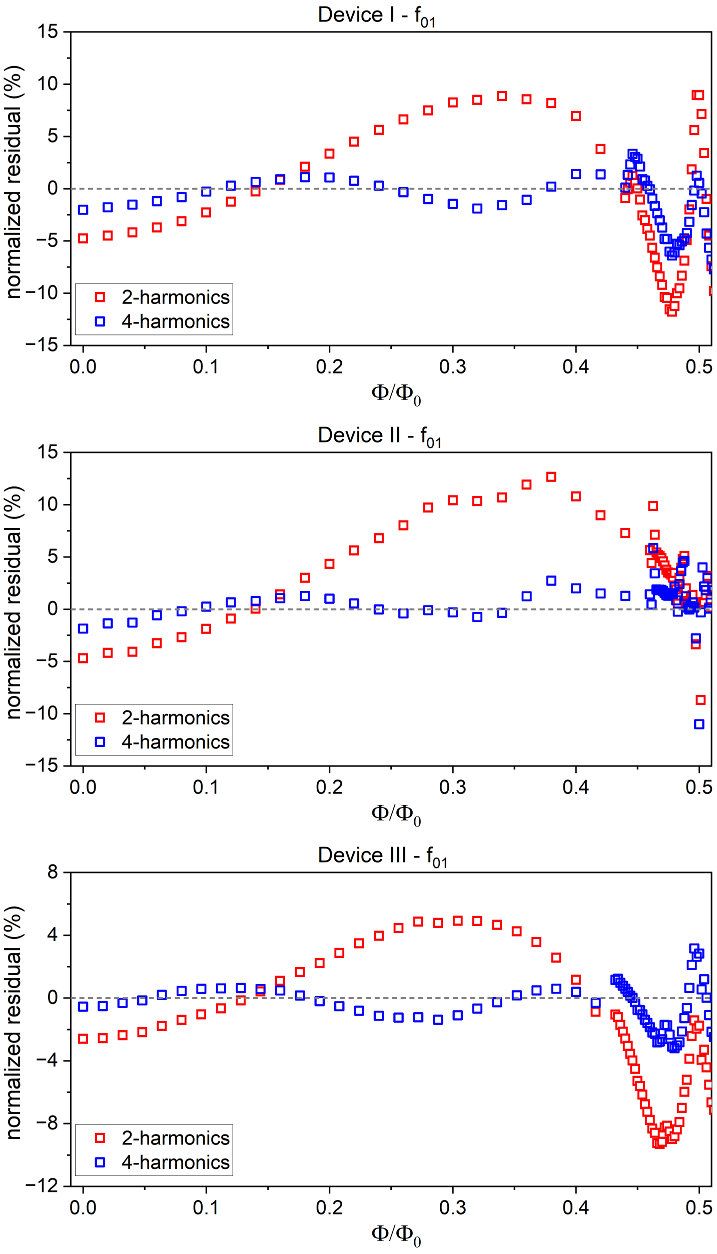

Supplementary Figure 5: Normalized residual $(f_{\mathrm{model}} - f_{\mathrm{meas}})/f_{\mathrm{meas}}$ as a function of Φ/Φ_0 for the $|0\rangle - |1\rangle$ qubit transition using the higher-harmonic model for device I, II and III, respectively. Here, k refers to the number of leading Fourier harmonic terms used to approximate the Josephson potential energy in $U = \sum_k E_{\mathrm{J}}^k \cos(k\varphi)$.

Supplementary Information 6: Multi-transparency model

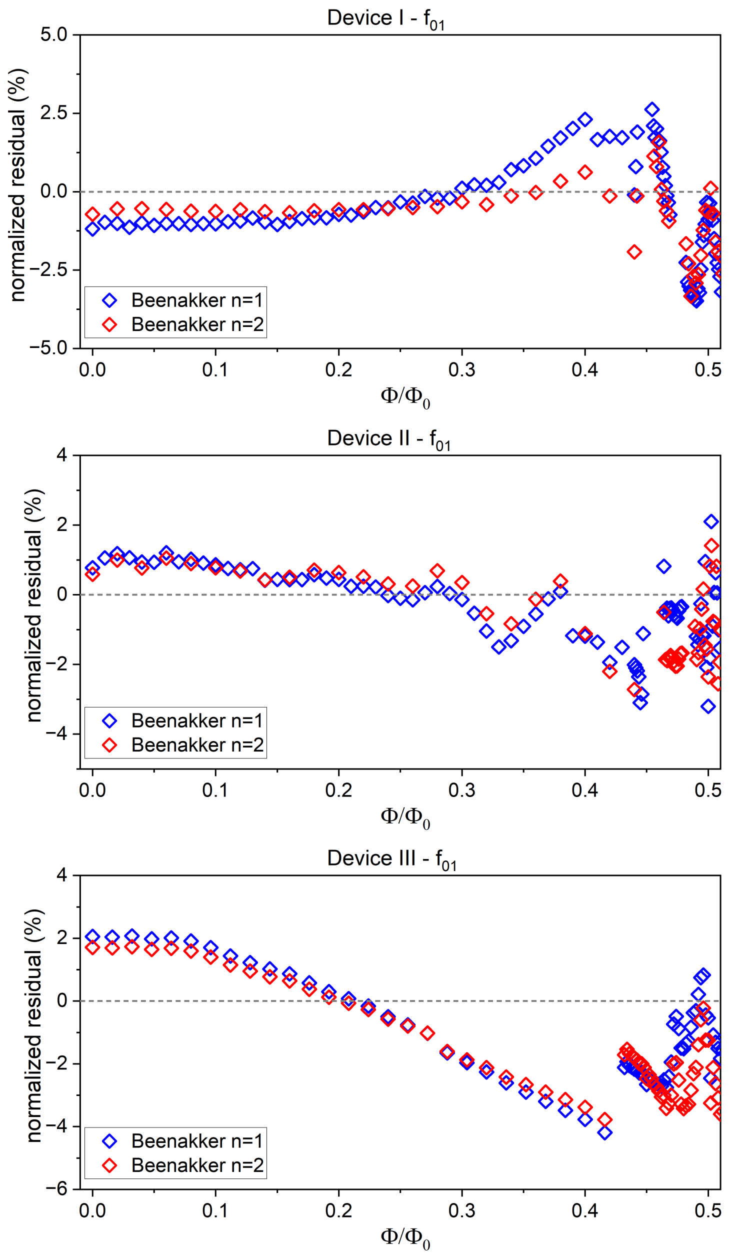

Supplementary Figure 6: Normalized residual $(f_{\mathrm{model}} - f_{\mathrm{meas}})/f_{\mathrm{meas}}$ as a function of Φ/Φ_0 for the $|0\rangle - |1\rangle$ qubit transition using the single and multiple transparency model for device I, II and III, respectively. Here, n refers to the number of characteristic channel transparencies for each Josephson junction. We observe that increasing n from a single ($n = 1$) characteristic channel transparency to two ($n = 2$) channel transparencies does not lead to any significant reduction in the residuals.

Supplementary Information 7: T_1 and T_2 flux dependence

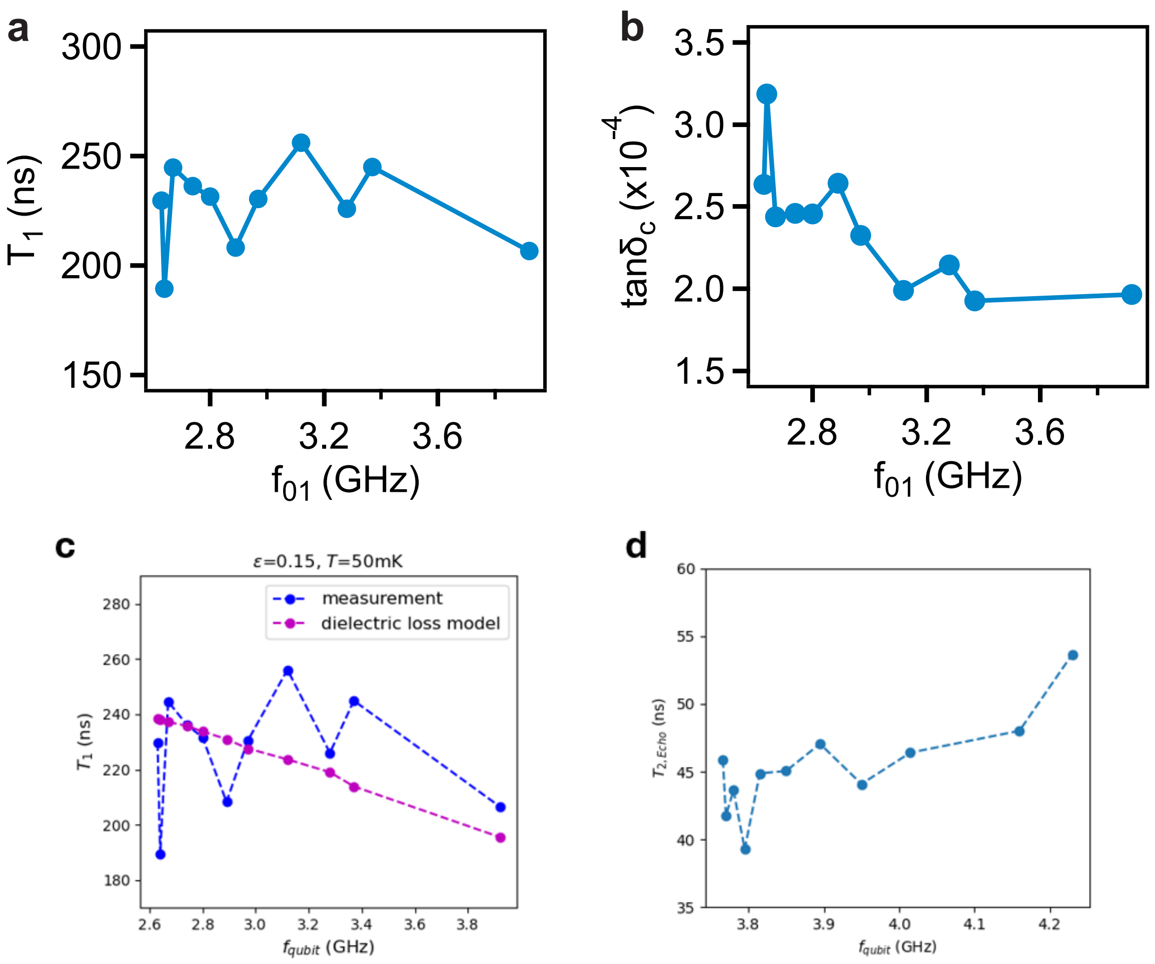

Supplementary Figure 7: **a** T_1 as a function of the $|0\rangle - |1\rangle$ transition qubit frequency f_{01} in the vicinity of half-flux quanta $\Phi = 0.5\,\Phi_0$. **b** The effective dielectric loss tangent $\tan\delta_c$ vs the qubit frequency f_{01} extracted from (**a**) using $\tan\delta_c = \frac{1}{T_1\omega_{01}}$, where $\omega_{01} = 2\pi f_{01}$. **c** T_1 as a function of the qubit frequency f_{qubit} around half-flux quanta $\Phi = 0.5\,\Phi_0$. The blue curve denotes the experimentally measured T_1, while the red curve denotes T_1 extracted from a dielectric-loss model. This model assumes a weak frequency dependence of the dielectric loss tangent given by: $\frac{\tan\delta(\omega)}{\tan\delta(\omega=2\pi\times2.63\,\mathrm{GHz})} = (\frac{\omega}{2\pi\times2.63\,\mathrm{GHz}})^{\varepsilon}$ with $\varepsilon = 0.15$. The reference value $\tan\delta(\omega = 2\pi\times2.63\,\mathrm{GHz}) = 2.44\times10^{-4}$ is typical for InP dielectric losses. **d** $T_{2,Echo}$ as a function of the qubit frequency f_{qubit} near $\Phi/\Phi_0 = 0.5$, measured in a separate cooldown, where the qubit frequency at $\Phi/\Phi_0 = 0.5$ is 3.76 GHz.

Supplementary Information 8: Vacuum Rabi Oscillation Fitting

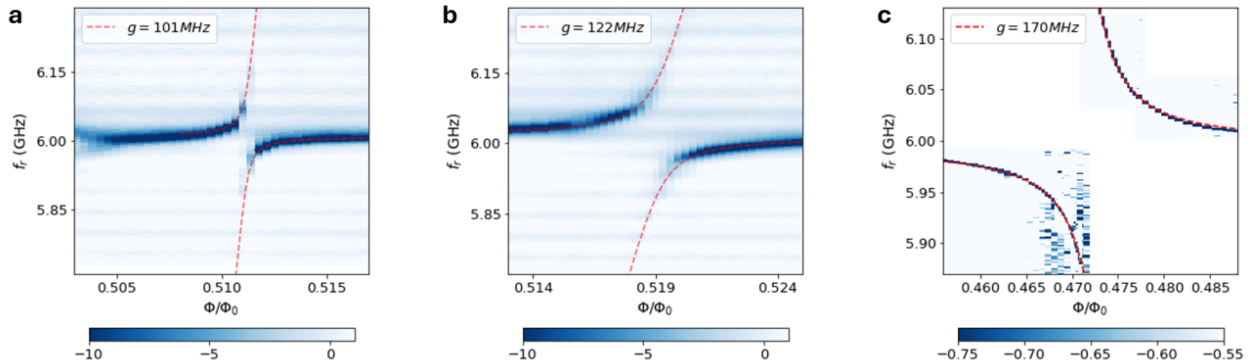

Supplementary Figure 8: **a-c** $|S_{11}|$ as a function of flux Φ/Φ_0 and resonator frequency f_{r} for device I, II and III, respectively. The red dashed lines show fits for the vacuum Rabi oscillation to extract the qubit-resonator coupling strength g. We note that $|S_{11}|$ is plotted in dB for device I (**a**) and device II (**b**), while figure (**c**) shows the normalized $|S_{11}|$ for device III.

In our work, we use the Jaynes-Cummings Hamiltonian denoted by:

$$H = \hbar\omega_{\ r} a^\dagger a + \frac{\hbar\omega_{ge}}{2}\sigma_z + \hbar g\left(a^\dagger\sigma_- + a\sigma_+\right)$$

where $a^\dagger$,a are the photon creation and annihilation operators, $\sigma_z, \sigma_\pm$ are the Pauli and ladder operators for the qubit, g is the vacuum coupling strength and $\omega_{\ r}$, $\omega_{\ ge}$ are the resonator and the qubit transition frequency respectively. The resultant shape of the avoided crossing is then given by:

$$E_{\pm,n} = \hbar n\omega_r \pm \frac{\hbar}{2}\sqrt{(4ng^2 + \Delta^2)}$$

where $\Delta = \omega_r - \omega_{ge}$ denotes the detuning of the qubit from the resonator frequency.

Supplementary Information 9: Single-Transparency Model Error Analysis

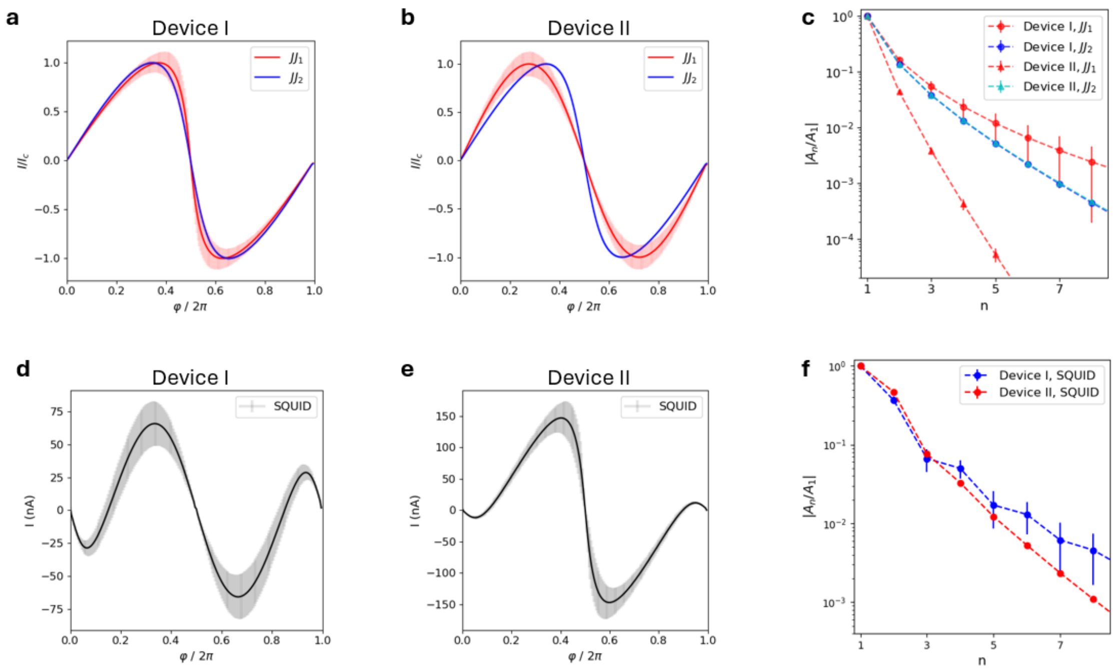

Supplementary Figure 9: **a,b** Current-phase relation (CPR) extracted from the single-transparency model for device I and II, with error bands indicating the 95% confidence intervals. We use the convention where we assign the notation JJ1 to the junction with larger critical current: I_{c1} = 178.5nA±19.7nA and I_{c2}=71.9nA±1.1nA for device 1, I_{c1}=138.0nA±17.6nA and I_{c2} = 100.8nA±1.2nA for device II. **c** The corresponding normalized harmonic amplitude $|A_n/A_1|$ and their error bars for each JJ in device I and II. **d,e** CPR of the SQUID loop at $\Phi/\Phi_0 = 0.5$ with 95% confidence interval error bands for device I and II. **f** The corresponding normalized harmonic amplitude $|A_n/A_1|$ and their error bars for the SQUID loops in device I and II.

We first determine the confidence intervals for the fitting parameters in table 1 by calculating the covariance matrix of the nonlinear least-squares fit. We follow White's heteroskedasticity-consistent method [H. White, Econometrica, vol. 48, no. 4, pp. 817–838 (1980)], where we first calculate the Jacobian matrix J of the fit numerically using a finite-difference scheme. The covariance matrix C is then approximated as:

$$C = \epsilon^2 \left(J^T J\right)^{-1}$$

where ϵ is the mean standard error of the fit. The 95% confidence interval for each fitted parameter β_i is then given by:

$$\beta_i \pm Z\sqrt{C_{ii}}$$

where $Z \approx 1.96$ is the Z-score that corresponds to the 95% confidence interval.

To propagate these uncertainties to the extracted CPR and the corresponding Fourier harmonics, we employ a bootstrapping scheme with 500 Monte-Carlo samples. Each parameter β_i is assumed to follow a normal distribution with standard deviation $\sigma = \sqrt{C_{i,i}}$, which is justified by the large number of data points relative to the number of fit parameters. Unphysical parameters, such as transmission exceeding unity, are excluded by post-selection. For each valid sample, the CPR is then computed numerically on a uniform grid of 200 phase points, and the harmonic amplitudes are extracted using a Fast Fourier Transform (FFT). The reported error bars for the current and the harmonic amplitude, as shown in supplementary figure 9, correspond to the median 95% interval of the resulting distributions.

Supplementary Information 10: Comparison of charge-matrix elements

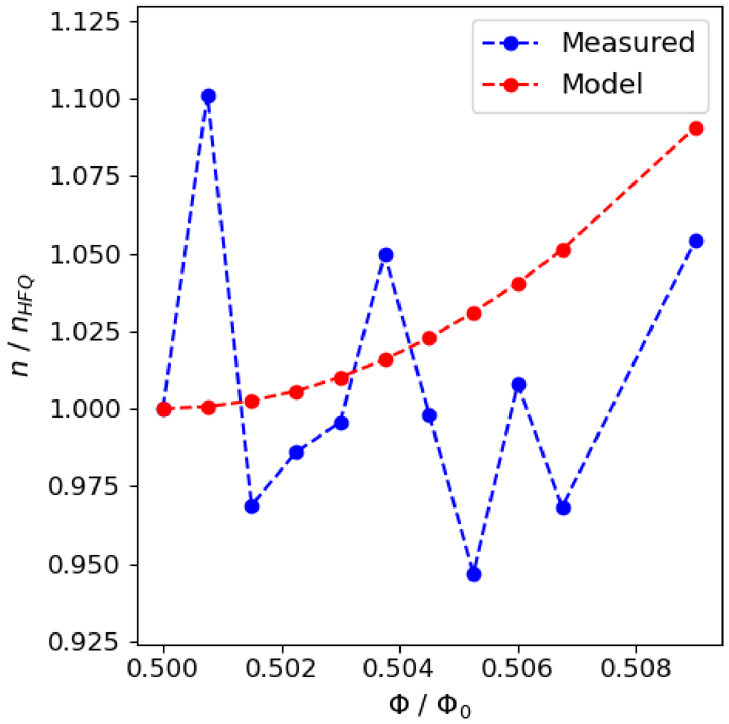

Supplementary Figure 10: Normalized charge matrix elements as a function of flux Φ/Φ_0 for device I, where the blue curve is extracted from the measured T_1 times in supplementary figure 7a using the relation: $1/T_1 \propto E_C |n_{01}|^2 \tan\delta$, where E_C is the charging energy, n_{01} is the charge matrix element and $\tan\delta$ is the dielectric loss tangent. The red curve is calculated using our single-transparency model,

Supplementary Information 11: Al coherence length and InAs mean-free path

The validity of the short-junction approximation is central to our modeling of the Andreev bound states (ABS) and the current-phase relation (CPR). In a superconductor-semiconductor-superconductor (S-Sm-S) junction, the short-junction limit is defined by:

$$L \ll \xi = \frac{\hbar v_F}{\pi \Delta}$$

where L is the JJ length, ξ is the superconducting coherence length, v_F is the Fermi velocity in the semiconductor and Δ is the induced superconducting gap. Using $\Delta \approx 230\,\mu$eV determined from the Al critical temperature of $T_c \sim 1.5\,$K [Yuan et al., J. Vac. Sci. Technol. A 39, 033407 (2021)] and $v_F \sim 10^6$ m/s corresponding to an electron density of $n_s \approx$ 7.68 X 10^{11}cm$^-$2, we estimate $\xi \sim 1\,\mu$m. For our junction length L=250 nm, this gives $\frac{L}{\xi} \sim 0.25$, placing our devices in the crossover of the short and intermediate regime.

The mean free path of the InAs 2DEG is given by:

$$l = \frac{\hbar k_F \mu}{e}$$

where $k_F = \sqrt{(2\pi n_s)}$ is the 2D Fermi wavevector, n_s is the electron carrier density and μ is the electron mobility. Using the measured values of $n_s \approx$ 7.68 X 10^{11} cm^{-2} and $\mu \approx 20,000\,$cm$^2/Vs$, we obtain $l \approx 290$ nm. Since the junction length is $L = 250$ nm, the ratio $l/L \approx 1.2$ places our device in the quasi-ballistic regime, but well away from the strongly diffusive limit ($l \ll L$). In this regime, many electron trajectories can traverse the junction without any scattering, giving rise to ballistic contributions to transport, while a non-negligible fraction will scatter, resulting in only a few effective high-transparency channels that dominate supercurrent transport. Although diffusive contributions to transport cannot be entirely excluded, the combination of length scales indicates that our junctions operate in the quasi-ballistic regime, consistent with transport picture presented in the manuscript.
